# Somatosensory Psychophysical Losses in Inhabitants of Riverside Communities of the Tapajós River Basin, Amazon, Brazil: Exposure to Methylmercury Is Possibly Involved

**DOI:** 10.1371/journal.pone.0144625

**Published:** 2015-12-11

**Authors:** Eliana Dirce Torres Khoury, Givago da Silva Souza, Carlos Araújo da Costa, Amélia Ayako Kamogari de Araújo, Cláudia Simone Baltazar de Oliveira, Luiz Carlos de Lima Silveira, Maria da Conceição Nascimento Pinheiro

**Affiliations:** 1 Núcleo de Medicina Tropical, Universidade Federal do Pará, Belém, PA, Brazil; 2 Instituto de Ciências Biológicas, Universidade Federal do Pará, Belém, PA, Brazil; 3 Secretaria de Saúde, Itaituba, PA, Brazil; 4 Universidade Ceuma, São Luís, MA, Brazil; Duke University, UNITED STATES

## Abstract

The purpose of this work was to evaluate the somatosensory system of methylmercury-exposed inhabitants living in the communities of the Tapajós river basin by using psychophysical tests and to compare with measurements performed in inhabitants of the Tocantins river basin. We studied 108 subjects from Barreiras and São Luiz do Tapajós, two communities of the Tapajós river basin, State of Pará, Amazon, Brazil, aged 13–53 years old. Mercury analysis was performed in head hair samples weighting 0.1–0.2 g by using atomic absorption spectrometry. Three somatosensory psychophysical tests were performed: tactile sensation threshold, vibration sensation duration, and two-point discrimination. Semmes-Weinstein 20 monofilaments with different diameters were used to test the tactile sensation in the lower lip, right and left breasts, right and left index fingers, and right and left hallux. The threshold was the thinner monofilament perceived by the subject. Vibration sensation was investigated using a 128 Hz diapason applied to the sternum, right and left radial sides of the wrist, and right and left outer malleoli. Two trials were performed at each place. A stopwatch recorded the vibration sensation duration. The two-point discrimination test was performed using a two-point discriminator. Head hair mercury concentration was significantly higher in mercury-exposed inhabitants of Tapajós than in non-exposed inhabitants of Tocantins (p < 0.01). When all subjects were divided in two groups independently of age—mercury-exposed and non-exposed—the following results were found: tactile sensation thresholds in mercury-exposed subjects were higher than in non-exposed subjects at all body parts, except at the left chest; vibration sensation durations were shorter in mercury-exposed than in non-exposed subjects, at all locations except in the upper sternum; two-point discrimination thresholds were higher in mercury-exposed than in non-exposed subjects at all body parts. There was a weak linear correlation between tactile sensation threshold and mercury concentration in the head hair samples. No correlation was found for the other two measurements. Mercury-exposed subjects had impaired somatosensory function compared with non-exposed control subjects. Long-term mercury exposure of riverside communities in the Tapajós river basin is a possible but not a definitely proven cause for psychophysical somatosensory losses observed in their population. Additionally, the relatively simple psychophysical measures used in this work should be followed by more rigorous measures of the same population.

## Introduction

The Minamata disease was the first well-investigated human syndrome related to mercury intoxication due to an environmental catastrophe [1,2]. It occurred after the consumption of contaminated fish or seafood by the inhabitants of Minamata and other villages in the Kumamoto Prefecture, Japan [2]. In addition to exposure through the diet, the occupational exposure to different forms of mercury compounds could also have serious health consequences and was also well documented [3–6]. Both types of exposure have the potential to damage the nervous system [7,8]. Based on accidents occurred in Japan and Iraq, the World Health Organization established that mercury concentration in the head hair above 50 μg/g was associated to a risk of 5% of neurological damage in adults [9].

In Minamata, neurological damage caused by methyl mercury exposure resulted in cognitive, motor, and sensory impairment, and a variety of neural symptoms of functional losses [10–15]. Somatosensory disturbances were crucial symptoms of Minamata disease [1]. Several aspects of the somatic sensory system disorders caused by mercury exposure were studied by using clinical, psychophysical, and electrophysiological quantitative procedures [8,14–17]. Inhabitants from Minamata and surrounding villages, exposed to mercury through the consumption of contaminated fish for about 20 years, showed paraesthesia of the extremities and lips even years after exposure withdrawal [1,18]. Some studies have suggested that mercury affected preferentially the somatosensory cortex instead of causing degeneration of peripheral nerves as initially suggested [19].

In the Amazon, some riverside communities were also exposed to mercury by fish consumption [20,21]. Mercury exposure of communities from the Tapajós river basin were attributed to intense gold mining activity in the region, making use of large quantities of mercury to extract the gold from the gold ore. Because of the associated risks of environmental contamination and human exposure, mercury levels in sediments, fish samples, and in the hair of the inhabitants of riverside communities were extensively monitored by several research groups that worked in the Amazon along the last three decades [20,22–36]. During this period, mercury-exposed communities from the Tapajós river basin showed mercury levels higher than non-exposed communities from other Amazon river basins, but their mean mercury level remained generally below those recommended to avoid neurological damage [34,36]. However, these low to intermediate levels of mercury exposure represent a form of chronic contamination that can be potentially dangerous to human health. The deleterious effects of chronic exposure to mercury on cognitive, motor, and sensory functions of inhabitants of Tapajós riverside communities was reported in several studies published in the last two decades [37–48].

Psychophysical measurements have been largely used as indicators of early neural damage caused by xenobiotic agents such as organic solvents, heavy metals, chloroquine and hydroxichloroquine, and others [49]. In comparison with neurological peripheral exams and the majority of electrophysiological procedures, psychophysical measurements can be used to evaluate the final outcome of sensory processes and the cumulative changes due to physical or chemical aggression that concurrently affect different levels of the sensory systems.

In this work, somatosensory functions of mercury-exposed inhabitants of the Tapajós river basin were evaluated by using psychophysical quantitative procedures. It was found that vibration sense, tactile sensation, and two-point discrimination were impaired in these populations. To our knowledge, this was the first work to perform this kind of evaluation in the Amazon. Thus, in this first series of measurements, we planned to replicate with mercury-exposed inhabitants of Amazon riverside communities similar study previously performed with Japanese patients from the Kumamoto Prefecture suffering from Minamata disease [7,8]. In addition, we tried to simplify the procedure and minimize the set of instructions as much as possible to avoid possible communication difficulties with the subjects tested. We feared that a more robust psychophysical procedure as those currently recommended in the literature [50,51] may pose difficulties for a quick assess of the target population.

The methods used are limited as it has previously being pointed out [50,51]. However, we tried to minimize these limitations both in the employed procedures and in the sober interpretation of results (see further below in Methods and Discussion). We are planning a sequel of this investigation in the same region using more sophisticated methods recommended in previous work of other research groups [50–53].

## Materials and Methods

### Communities

**Fig 1:**
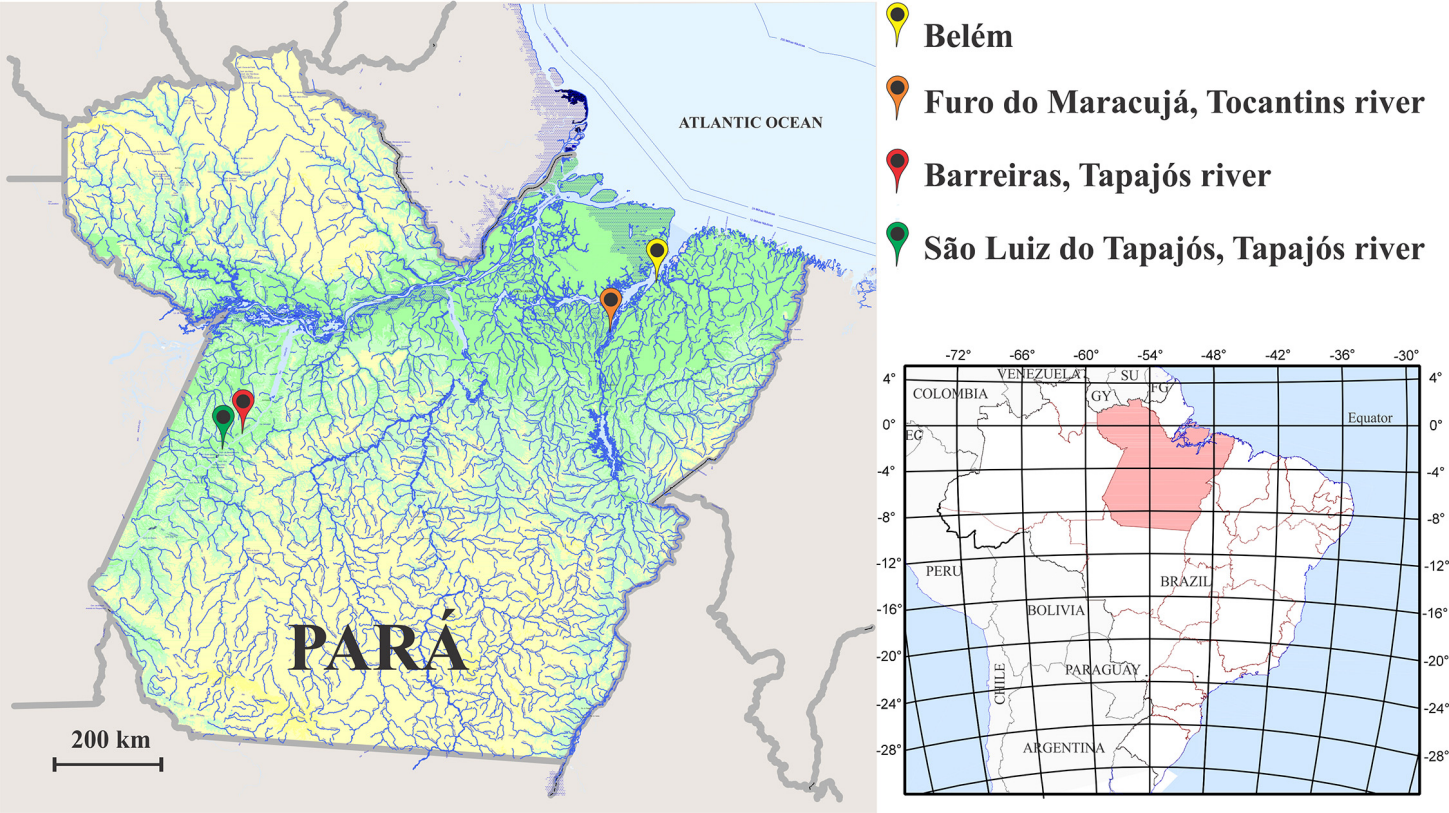
State of Pará, Brazil. The somatosensory functions of inhabitants from two communities of the Tapajós river basin, Barreiras and São Luiz do Tapajós, and one community of the Tocantins river basin, Furo do Maracujá, were studied with psychophysical methods. Barreiras and São Luiz do Tapajós were mercury-exposed due the proximity of gold-mining areas. The map was obtained from the Instituto Brasileiro de Geografia Estatística–IBGE. Modified from: ftp://geoftp.ibge.gov.br/mapas_tematicos/fisico/unidades_federacao/pa_fisico.pdf

Three riverside communities from two different regions of the State of Pará, Brazilian Amazon, were studied (Fig 1). All three communities had similar diets rich in fish consumption [31]. The first region was located in the Tapajós river basin, a region suffering the influence from gold-mining activity. In the process of amalgamation with gold followed by heating to obtain the precious metal, mercury is released in the environment, deposited in river sediments, converted to methyl mercury, and incorporated to the food chain of the riverside population of Tapajós. Two Tapajós communities were studied: São Luiz do Tapajós, 04° 20’ 31‘’ S, 56° 15’ 02” W; and Barreiras, 04° 05’ 52” S, 55° 40’ 59” W.

The second region was elected as a control area and it was located in the Acará River, a subsidiary of the Tocantins river basin, a region without any influence of gold-mining activity. In this region, we studied the community of Furo do Maracujá, 01° 32’ 07” S, 48° 29’ 26” W.

### Subjects

We studied 108 subjects from Tapajós and 49 subjects from Tocantis, respectively. All subjects were between 13–53 years old (Tapajós: 33.2 ± 11.2 years old, 77.8% females and 22.2% males; Tocantins: 30.5 ± 11.8 years old, 63.3% females and 36.7% males). The mercury-exposed subjects and control subjects were matched for age, gender, and social-economic profile. The inclusion criteria were to be living at least two years in the community, no additional diseases with nervous system involvement, and no present or past jobs related to gold-mining activity.

### Ethics statement

All subjects were informed about the aims and procedures of the present study. All subjects provided written consent to participate in this study. The parents or guardians gave a written consent on behalf of children or adolescents. The procedures obeyed the tenets of the Declaration of Helsinki and Resolution 196/96 of the National Council of Health, Ministry of Health, Brazil. The research was approved by the Research Ethics Committee of the Tropical Medicine Nucleus, Federal University of Pará, Belém, State of Pará, Brazil, Proc. #46/2007.

### Mercury analysis

Total mercury content was determined in 10–20 mg head hair samples, preferentially from the occipital region, and 1 cm above the hair insertion on the head. They were analysed using cold-vapour atomic absorption spectrometry (Mercury Analyzer HG-201, Sanso Seisakusho, Tokyo, Japan) in the Human Toxicology Laboratory of the Federal University of Pará (Belém, Pará, Brazil), following the protocol of Suzuki and colleagues [54]. The analytical quality control was warranty by the International Atomic Energy Agency certification (IAEA-085). Duplicate sample analyses were performed and the results were shown in μg/g. The methodology validation for total Hg determination was performed using reference human hair material (IAEA-086). The detection limit (DL) for total Hg determination was 0.001 mg.kg^-1^ and the quantification limit (QL) was 0.010 mg.kg^-1^.

### Somatosensory psychophysics

All evaluations were part of the routine clinical exam of inhabitants of Amazon communities and were performed in the community where they resided. The general feeling was that subjects had a genuine motivation to faithful collaboration with the exams. It was not possible to take the subjects to be tested by neurologists who did not know where subjects lived and the risks they were subjected due to mercury exposure. The experimenter bias was thus difficult to overcome, once it was not possible to perform a double-blind experiment in such environment and circumstances. All procedures were performed in a room with temperature kept between 26–29°C.

Somatosensory psychophysical tests comprised measurements of tactile sensation threshold, vibration sensation duration, and two-point discrimination threshold at several body locations [8]. While other studies have employed two or more neurologists to exam the subjects [8], in this study the same neurologist performed all exams. The difficult access to the communities and the long period needed to stay in the communities limited the number of physicians available to perform for the neurological exam in such conditions. Subjects were extensively instructed about each test prior measurements. The set of instructions was rigorously kept the same throughout the series of mercury-exposed subjects and non-exposed subjects. Subjects did not receive feedback after each trial during any of the three tests in attempt to keep them attending the initial instructions. For sake of simplicity, it was avoided the use of sophisticated psychophysical methods such as a two-interval, forced-choice procedure.

Tactile sensation thresholds were estimated using a battery of 20 different Semmes Weinstein monofilaments of the same length (38 mm) and different diameters, 1.65 to 6.65 mm, buckling force ranging from 0.0045 to 447 g, which is also the force felt by the patient when the monofilament bends. Following the ascending order of buckling force, monofilaments were applied on the lower lip non-pigmented region, right and left upper breasts at 5 cm from the midline bellow the clavicle, and volar surface of the right and left index and hallux fingers [8]. During the test, subjects were in dorsal decubitus keeping eyes closed. Each monofilament was pressed against the skin during 1 s and then removed, and subjects had to indicate if they had felt the tactile sensation and the place where the pressure was applied. One trial was performed for each subject. The thresholds were taken as the log of the buckling force of the thinner monofilament detected by the subject at each body location after three consecutive trials. A threshold of 500 g was assigned at a body location when the subject did not detect even the thickest monofilament (buckling force of 447 g, size 6.65 mm) in that region.

Vibration sensation duration was evaluated using a 128 Hz tuning fork. The subject rested in dorsal decubitus and the tuning fork was applied perpendicularly to the upper region of the sternum, radial side of the right and left wrist, and right and left outer malleolus. When the tuning fork was applied, the neurologist started a stopwatch. The subject had to report when the vibration sensation ceased, and at this point the neurologist stopped the watch in order to estimate the duration of the vibration sensation. Each body location was tested twice and the sensation duration was taken as the average of both trials.

Two-point discrimination thresholds were evaluated using a plastic aesthesiometer device. Lower lip and volar surface of the right and left index fingers were tested [15]. Subjects kept their eyes closed and were instructed that one or two ends of the aesthesiometer would touch them in a random sequence. The neurologist equally pressed subjects’ skin for 1 s in different trials. Immediately after detecting the touch, subjects had to indicate the number of points they have been touched–one or two. For each right response, the two-point distance was increased by 1 mm. The threshold was taken as the minimum two-point distance detected by the subjects in three consecutive trials.

Measurements of two-point discrimination thresholds evaluated in this way are influenced by intensity and spatial cues [50,51]. Subjects were extensively instructed about the criterion to use in the choice of one or two points and to avoid judgements based on intensity criteria.

### Statistical analysis

We performed an observational, analytical, case-control study. Total mercury concentration and psychophysical performance from mercury-exposed and non-exposed subjects were compared using Student’s t-test with Bonferroni multiple-significance-test correction (p-value = 0.05/n, n = number of comparisons). In order to evaluate the dependence the psychophysical parameters on the mercury concentration, we investigated the Pearson product-moment correlation coefficient (r) between psychophysical thresholds and mercury concentration. In addition, we investigated how the cumulative psychophysical mean performance varied as a function of mercury concentration.

## Results

### Mercury levels

**Fig 2:**
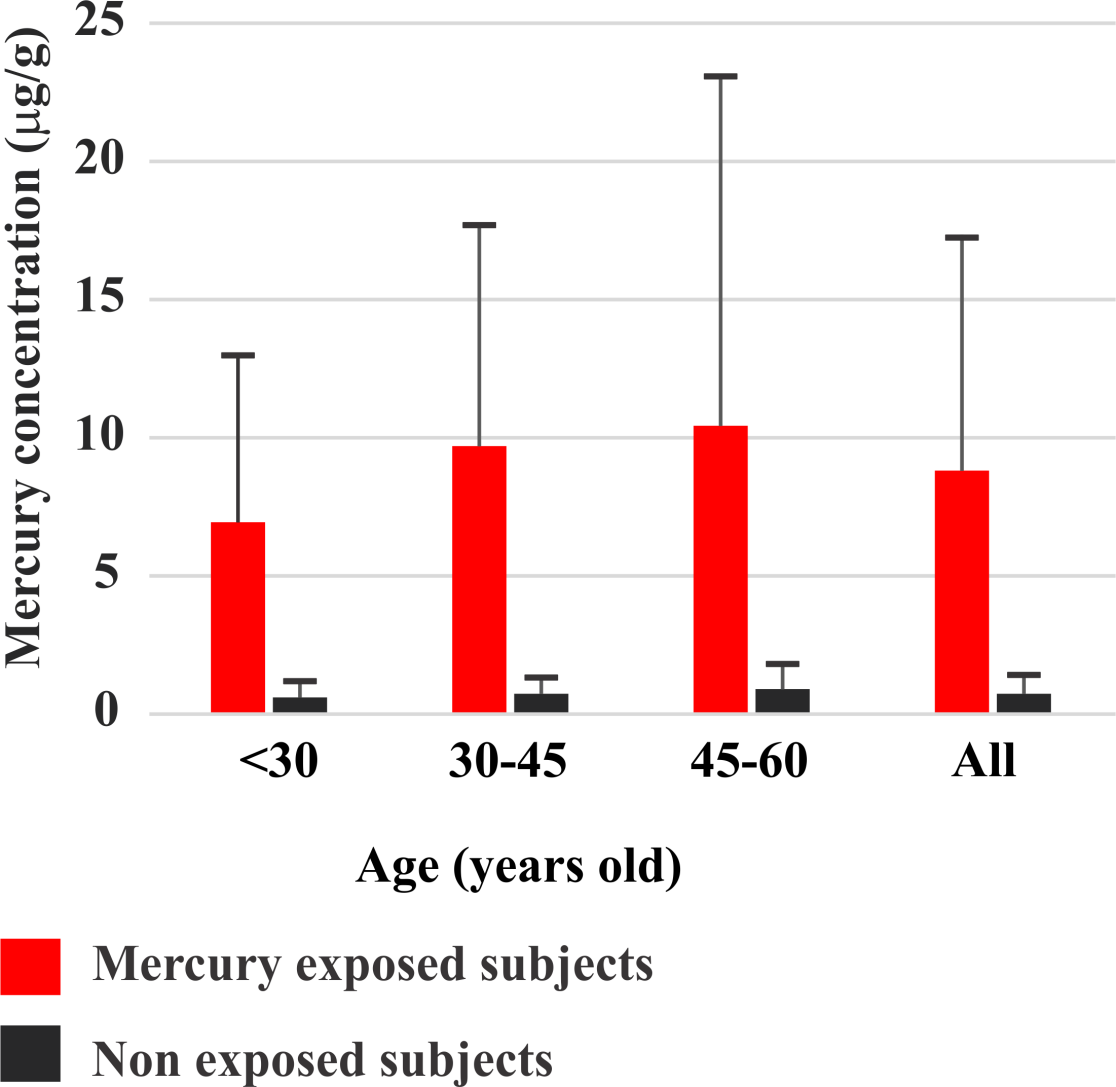
Mercury concentration in Amazon riverines. Comparison of the total Hg concentration found in two population of the Amazon: mercury-exposed inhabitants of the Tapajós river basin (red bars) and non-exposed inhabitants of the Tocantins river basin (black bars). Subjects were grouped by age. The rightmost bars grouped all subjects independently of age. Due to the influence of gold mining activity, inhabitants of the Tapajós have significant higher mercury levels than inhabitants from the Tocantins. In addition, mercury accumulates with age, reflecting the longer exposure to the metal.

In the Tapajós river communities, the total Hg concentration in the head hair samples ranged between 0 and 60 μg/g (mean ± standard deviation: 8.8 ± 8.53 μg/g; n = 108), while in the non-exposed community ranged from 0 to 2.4 μg/g (mean ± standard deviation: 0.73 ± 0.59 μg/g; n = 49). The total Hg concentration in head hair samples from Tapajós was significantly higher than those from Tocantins (p<0.05). In the mercury-exposed areas, one subject had total Hg concentration above 50 μg/g and 28 subjects between 10 and 50 μg/g. In the non-exposed areas no subject had mercury levels above 2.4 μg/g. Fig 2 illustrates the differences in mercury levels observed in inhabitants of the Tapajós and Tocantins per age groups. It is noticeable that mercury levels significantly increase with age in the Tapajós reflecting a longer exposure to the metal that occurs along the life. There was also an increase of the small mercury levels observed in the non-exposed population of the Tocantins that not reached statistical significancy.

### Somatosensory psychophysical results

**Fig 3:**
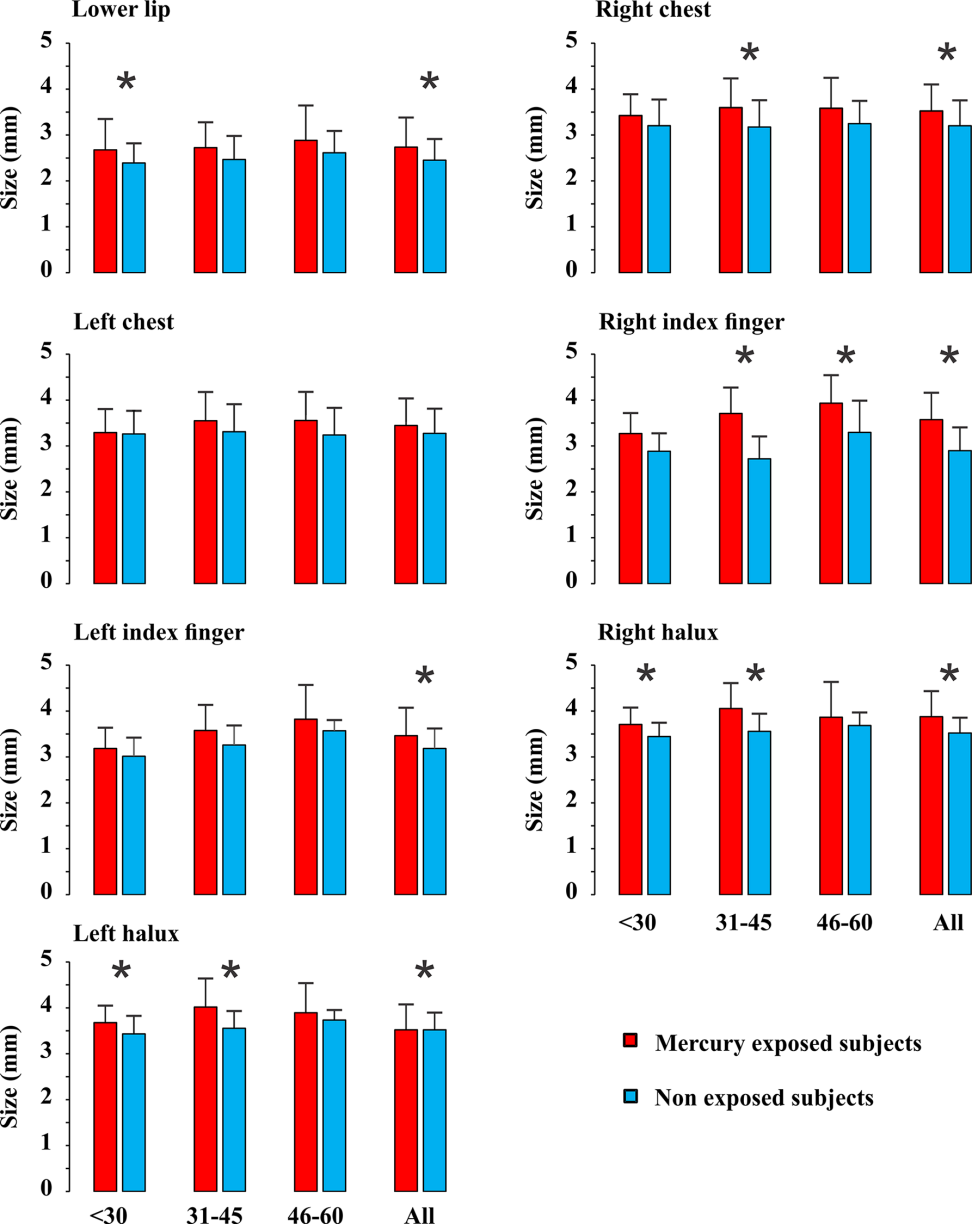
Tactile sensation thresholds. Measurements were performed at different body locations. Results are presented for different age groups, < 30, 31–45, 46–60 years old, and also taken together all subjects independently of age. Comparisons were made between mercury-exposed subjects and non-exposed subjects from three different Amazonian villages. Thresholds were expressed as the diameter of the filament probe and were higher at all body locations in all mercury-exposed groups when compared with non-exposed groups, but only reached statistical significance (p<0.05) in 11 out of 21 different conditions (*). When data from all age groups were pooled together, differences between mercury-exposed subjects and non-exposed subjects reached the significance level in six out of seven body locations (*).

**Fig 4:**
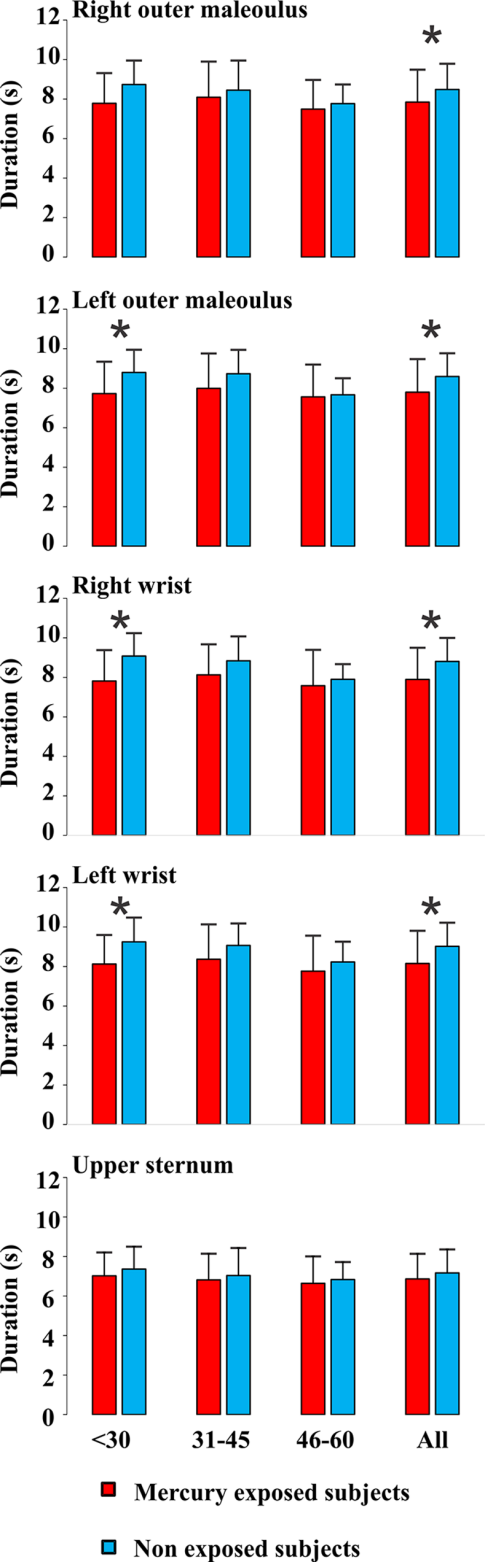
Vibration sensation durations. Measurements were performed at different body locations. Results are presented for different age groups, < 30, 31–45, 46–60 years old, and also taken together all subjects independently of age. Comparisons were made between mercury-exposed subjects and non-exposed subjects from three different Amazonian villages. Durations were shorter at all body locations in all mercury-exposed groups when compared with non-exposed groups, but only reached statistical significance (p<0.05) in two out of 15 different conditions (*). When data from all age groups were pooled together, differences between mercury-exposed subjects and non-exposed subjects reached the significance level in four out of five body locations (*).

**Fig 5:**
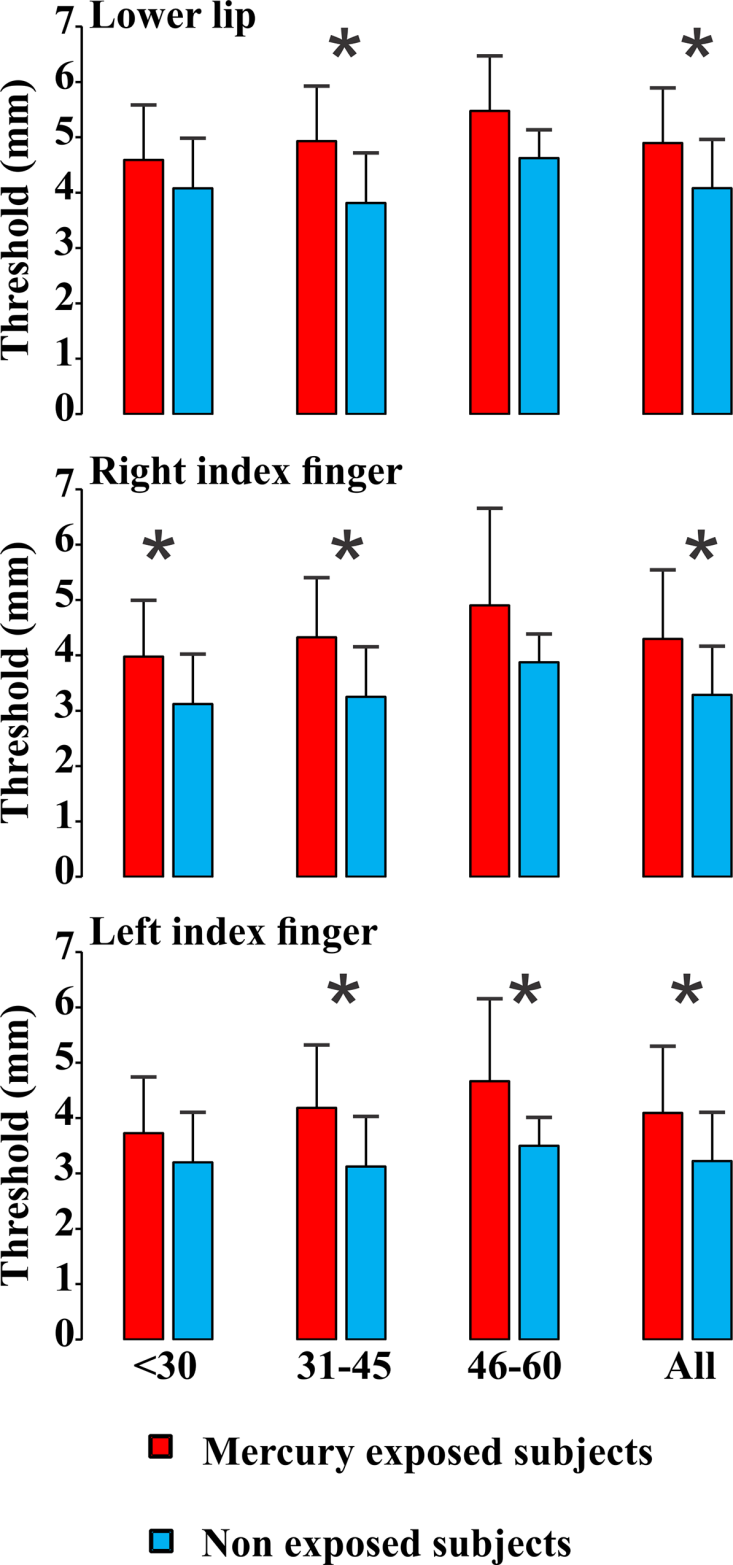
Two-point discrimination thresholds. Measurements were performed at different body locations. Results are presented for different age groups, < 30, 31–45, 46–60 years old, and also taken together all subjects independently of age. Comparisons were made between mercury-exposed subjects and non-exposed subjects from three different Amazonian villages. Thresholds were higher at all body locations in all mercury-exposed groups when compared with non-exposed groups, reaching statistical significance (p<0.05) in five out of nine different conditions (*). When data from all age groups were pooled together, differences between mercury-exposed subjects and non-exposed subjects reached the significance level in all three body locations that were tested (*).

Figs 3, 4, and 5 show the distribution of the results for mercury-exposed and non-exposed subjects either grouped independently of age (columns labelled “All” in each histogram) or divided in age groups (columns in the left side in all histograms). For all subjects grouped in a single group independently of age, there were significant statistical differences for the three measurements at most of body locations investigated (p<0.05). Tactile sensation thresholds estimated from Tapajós subjects were significantly higher than those estimated from Tocantins subjects at six out of seven body locations that were tested (Fig 3). Vibration sensation duration was shorter in Tapajós subjects than Tocantins subjects at four out of five body locations tested (Fig 4). Two-points discrimination thresholds were significantly higher in Tapajós subjects than in Tocantins subjects at all three body locations that were tested (Fig 5).

When subjects were divided in age groups, smaller proportions of significant differences were observed: 8/21 for tactile sensation thresholds, 3/15 for vibration sensation duration, and 5/9 for two-points discrimination thresholds. In all comparisons, however, mercury-exposed subjects performed worst than non-exposed subjects, suggesting that the division of subjects in small groups may have contributed for the lack of statistical significance of the observed differences.

In addition, Figs 3–5 also illustrate the relationships between somatosensory performance and age in the two studied groups, mercury-exposed and non-exposed subjects. For the two groups, the differences between the age classes were not statistically significant for all the three somatosensory tests. However, there was a small tendency for the tactile sensation thresholds increase, vibration sensation durations decrease, and two-points discrimination thresholds increase with age for both mercury-exposed and non-exposed subjects.

### Correlation between somatosensory measurements and the total mercury concentration in mercury-exposed subjects

There was no significant linear correlation between two-point discrimination threshold or vibration sense duration measured at any of the body locations tested in this work and total mercury concentration in the head hair of mercury-exposed subjects. On the other hand, there was a weak significant linear correlation between tactile sensation thresholds and total mercury concentration at all locations tested: lower lip, r = 0.23; right chest, r = 0.23; left chest, r = 0.2; right index finger, r = 0.2; left index finger, r = 0.22; right hallux, r = 0.26; and left hallux, r = 0.23.

**Fig 6:**
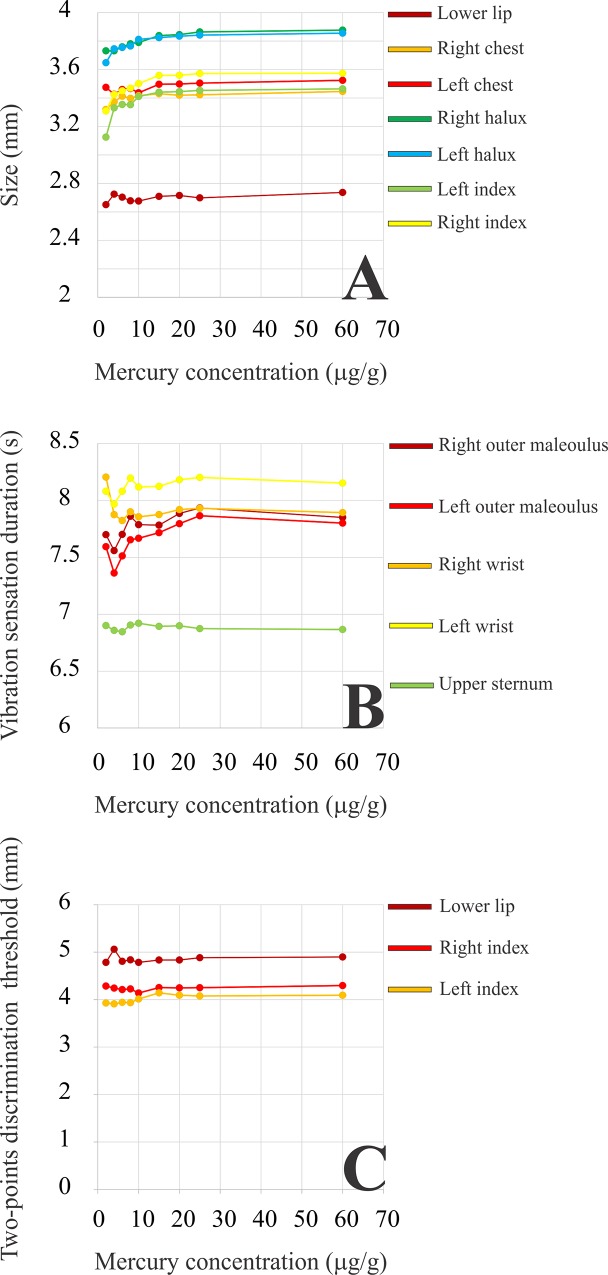
Tactile sensation thresholds, vibration sensation durations, and two-points discrimination thresholds–cumulative means. Cumulative means for tactile sensation thresholds, vibration sensation durations, and two-points discrimination thresholds as functions of the concentration of total Hg in the head hair for the mercury-exposed population of the Tapajós river basin. Each point represents the mean psychophysical performance for subjects with less than or equal to a mercury concentration. For the majority of body locations tested, tactile sensation thresholds increase and vibration sensation durations decrease as functions of the mercury level. There were no systematic changes of two-points discrimination thresholds with mercury level.

For the group of mercury-exposed subjects, it was estimated the mean cumulative psychophysical performance in the three tasks as a function of mercury concentration in the head hair (Fig 6A–6C). The cumulative mean represents the average psychophysical performance for all subjects with lower than or equal to a certain mercury concentration. For the tactile sensation threshold (Fig 6A) and vibration sensation duration (Fig 6B), it was found that for the majority of regions of the body, the somatosensory performance initially decreased with the level of mercury exposure and then remained at this level up to the highest levels of mercury found in the population examined. For two-points discrimination thresholds, there were no systematic changes of somatosensory performance as a function of mercury exposure.

## Discussion

Somatosensory disturbances were among the first symptoms reported by subjects exposed to toxic doses of methylmercury [3,4]. In Minamata disease, somatosensory impairment occurs before visual, auditory, and motor disturbances [16]. Previous studies performed in patients from Minamata and neighbouring villages exposed to methylmercury through fish consumption, described the toxic effects of mercury on patients’ somatosensory functions [7,8,15].

In spite of the relevance of somatosensory symptoms for the diagnosis of mercury intoxication, previous studies aimed to evaluate sensory functions of inhabitants of Tapajós focused on the exam of the visual system and reported that visual field extension, contrast sensitivity, and colour vision were impaired in these subjects [37,38,44,48]. In a previous study, it was reported the results of routine neurological exam performed in mercury-exposed subjects from the Tapajós which included somatic sensory exam [47]. To our knowledge, this work was the first extensive investigation of somatosensory functions of inhabitants of riverside communities of the Brazilian Amazon employing psychophysical methods in the same way as those recently used to study of patients suffering from Minamata disease [7,8,15].

In this study, it was compared the somatosensory performance of subjects living in Amazon riverside villages with or without story of mercury exposure. We studied communities from different hydrographic regions–Tapajós river basin and Tocantins river basin. Barreiras and São Luiz do Tapajós are communities with well documented exposure to mercury released from gold-mining activity [21,29–33,36–38,55]. The mercury exposure of the Tapajós river communities was closely monitored along the last two decades [36], and it was found that mercury exposure was decreasing, but many subjects (26.85%) still showed mercury concentration in the head hair above the tolerable levels [9].

The somatosensory examination is very important to detect methylmercury chronic effects [7,8,15]. Our findings showed that the tactile sensation thresholds, vibration sense duration, and two-point discrimination thresholds were impaired in inhabitants of the Tapajós river basin chronically exposed to methymercury through fish consumption in comparison with inhabitants of a non-exposed, control community from the Tocantins river basin with similar diet and socioeconomic profile [31].

**Fig 7:**
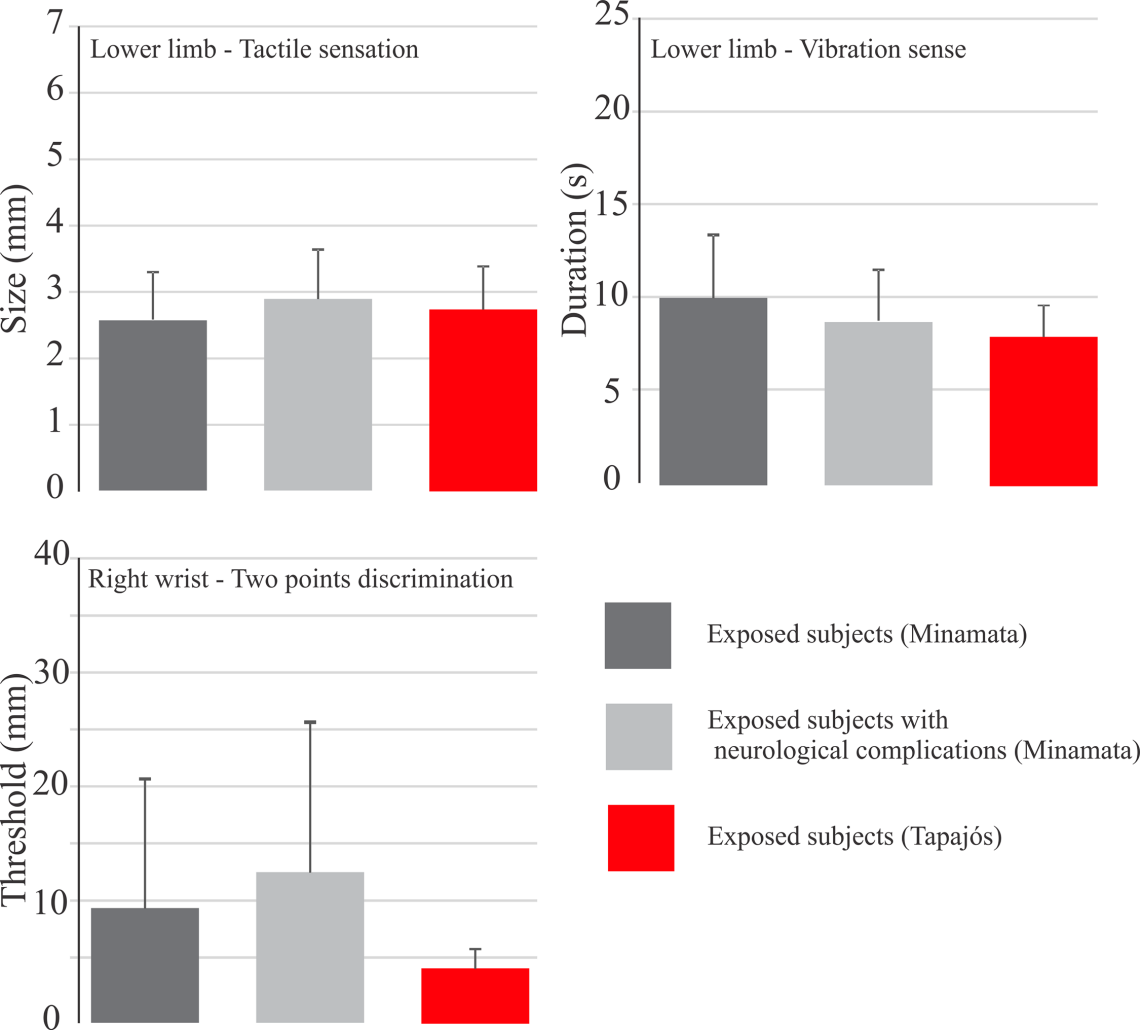
Comparison between somatossensory findings in Minamata and Tapajós. Comparison of the results of three different measurements–tactile sensation thresholds, vibration sensation durations, and two-points discrimination thresholds–obtained from mercury exposed subjects in the Minamata region (Japan) and Tapajós river basin (Brazilian Amazon). One body region for each measurement was chosen for illustration purposes. Minamata subjects were studied by Takaoka and colleagues [8] and divided in two groups: mercury-exposed subjects and mercury-exposed subjects with neurological complications. Tapajós subjects were studied in the current work and had no neurological complications as described for Minamata subjects. Tactile sensation thresholds in the lower limb was similar in the three groups. Vibration sensation durations were lower in the Tapajós subjects than in the Minamata subjects without neurological complications. Two-points discrimination thresholds in the right wrist were much higher with larger variability in Minamata subjects when compared with Tapajós subjects.

Impairment of somatosensory modalities was also found in Japanese populations exposed to methylmercury and suffering from Minamata disease [8,15]. Takaoka and colleagues found impairment of tactile sensation thresholds estimated from the chest were abnormally higher than thresholds estimated from the index and hallux [8] while Ninomiya and colleagues found a similar impairment in distal limbs, proximal limbs, and trunk in mercury-exposed subjects [15]. Takaoka and colleages also found impairment of vibration sense duration in mercury-exposed subjects [8]. Two-point discrimination thresholds were also found altered in mercury-exposed subjects of Minamata and Iraq [8,10,15,18]. The impairment of two-point discrimination thresholds in mercury-exposed patients reported suffering from Minamata disease was more severe than that reported in this study for equivalent body locations [8,15] (Fig 7).

Most of the differences observed in the results reported in this study of Amazon populations and in previous studies of Japanese populations may be related to the level of mercury exposure and subjects’ age. In the studies performed in Japan, patients were initially exposed to very high levels of methylmercury, much higher than those observed in the Amazon. In addition, the subjects from the Amazon that were studied in this work were generally younger than those investigated in the Japanese studies.

It was observed that only the tactile sensation thresholds were linearly correlated to the total mercury concentration in the head hair. Although there are no evidences in the present study that all the somatosensory modalities impairments have an association with the current level of mercury exposure, it is not possible to exclude the hypothesis that somatosensory losses reflect the long-term mercury exposure by fish consumption as observed previously [29,37,38]. There is no agreement in the literature about the linear correlation between methylmercury concentration and any functional deficit in riverine population, but many studies reported functional losses in mercury-exposed population in Amazon [37–48]. The interpretation of the findings of this work follows the same rational of the interpretation of results previously obtained in the above-mentioned studies of similar communities of the Amazon.

All three psychophysical somatosensory measurements performed in the inhabitants of Amazon villages have been widely used in several studies about nervous system impairment due to many causes including mercury poisoning, as mentioned in the previous paragraphs. However, the interpretation of results should be approached with caution [51–53]. A first reason to be cautious is that a more rigorous psychophysical approach could be desirable, including the use of tasks such as two-interval forced-choice, associated with a preselect series of stimulus presentation (methods of constants) or more elaborate methods of stimulus presentation such as Bayesian or conventional staircase adaptive presentations [51–53]. The choice of such tasks and methods could be employed to prevent response bias due to different causes, and the analysis of results could be set to a very precise conversion of the data collected during the experiment into measurements such as thresholds [56]. In this first series of such measurements performed in the Amazon, it was avoided the use of more sophisticated psychophysical procedures for two reasons. First, it was planned to replicate with the Amazonian population, similar study previously performed with Japanese patients suffering from Minamata disease [7,8]. Second, it was attempted to simplify the procedure and to minimize the set of instructions as much as possible to prevent possible difficulties to convey the purpose and procedure of the measurements to the subjects tested. We feared that a more robust psychophysical procedure could pose difficulties for a quick assess of a large number of subjects in the short time available to perform measurements.

A second reason to be cautious is that subject responses in the two-point threshold measurements could be based on intensity cues for very small point separations and then switched to spatial cues for large point separation. This may turn two-point threshold measurements, as performed in this work, a doubtful quantification of spatial acuity [50,53]. In this work, it was attempted to avoid the use of intensity cues by extensive instructions delivered to the subjects before the start of measurements. During the measurements themselves, there was no feedback to the subjects. Indeed the two-point threshold values reported in this work for non-exposed subjects were more than twice as large as those reported in the literature for normal subjects using different procedures [51,52]. We do not know why the threshold values were so higher than those reported in the literature. Different habits of work or environment climate may collaborate to this difference. In addition, subjects could have avoided any intensity cues and reported the difference between one and two points only when two points were clearly discernible, in compliance with the instructions they received and as planned in the experimental design. The threshold values reported seem to be in agreement with previous reports of the amount of point separation needed for subjects to abandon intensity cues in favour of spatial cues in the two-point threshold task as performed in this work [51].

Sensory impairment in methylmercury or metallic mercury toxicity has been related both to peripheral damage of somatic sensory nerves, auditory nerves, and retinal neural cells, and to damage of primary sensory cortical areas. This has been supported by a variety of anatomical-pathological studies that examined post-mortem tissues from patients that suffered from different forms of mercury poisoning [3,4,19,57–59]. In visual system, it has showed that pathological changes both in the anterior portion of the calcarine gyrus [4,57–59] and the central retina [60] can be related to the constriction of the visual field–a cardinal symptom of Minamata disease–and the contrast sensitivity and colour vision losses of patients exposed to metallic and organic mercury. Some body regions specialized in somatic sensory information such as the lips and fingers tip are characterized by increases receptor density, high number of dedicated pathways linking the periphery to the high nervous stations, and high metabolic activity along the regions that process their information. It would be fascinating to dissect the peripheral and central effects of mercury toxicity along the somatic sensory pathway for different regions of body representation, but this outside the scope of this work and must be reserved for its sequels.

## Conclusions

Long-term mercury exposure of riverside communities in the Tapajós river basin is a possible but not a definitely proven cause for psychophysical somatosensory losses observed in their population. Additionally, the relatively simple psychophysical measures used in this work should be followed by more rigorous measures of the same population.

A stronger case in favour of mercury toxicity as the likely cause of the somatosensory losses observed in the Tapajós population could be established if the current study had found a clear relationship between levels of mercury and sensory results. It would be unlikely that experimenters and subjects were aware of subject’s level of mercury at the time of psychophysical testing. That being the case they could not consciously or unconsciously shift their criteria during testing. However, no matter how decisive a strong relationship between increasing levels of mercury and declining sensory performance could be for this and similar studies, the fact is that it has not been demonstrated that such a simple relationship between these two measurements exists. In the current work, the results were mixed: a weak significant effect with tactile sensation thresholds and a non-significant trend with vibration sensation durations, and no trend with the two-point discrimination thresholds.

We have already acknowledged that this study is a first step to understand the relationship between mercury toxicity and somatosensory disturbance in the Amazon. More sophisticated procedures, certainly more cumbersome, time consuming, and difficult to carry out, will be part of further studies. In spite of the difficulties in carrying out such work, it could be done if resources are available, as it had been done before in the investigation of the effect of mercury toxicity on the visual system by several research groups.

## Supporting Information

S1 Dataset Mercury concentrations. **Psychophysical measurements.** Values obtained from mercury-exposed subjects and non-exposed subjects from Amazon riverside villages.
(XLSX)
